# Complex clinical case with Class III and open bite: stability after seven years

**DOI:** 10.1590/2177-6709.25.2.032-043.oar

**Published:** 2020

**Authors:** Rhita Cristina Cunha Almeida, Livia Kelly Ferraz Nunes, Ingrid Balbino Sousa Coelho Vieira, Felipe de Assis Ribeiro Carvalho, Marco Antonio de Oliveira Almeida

**Affiliations:** 1 Universidade do Estado do Rio de Janeiro, Faculdade de Odontologia, Departamento de Ortodontia (Rio de Janeiro/RJ,Brazil).

**Keywords:** Stability, Open bite, Class III

## Abstract

A healthy 15-year-old boy with anterior open bite, edge-to-edge transverse discrepancy, and Class III skeletal relationship sought a nonsurgical orthodontic treatment. The patient was treated with premolars extraction, a Hyrax expander and intrusion mechanics with vertical elastics. This mechanics allowed for excellent facial and occlusal results. The final occlusion presented Class I molar and canine relationships, ideal overjet and overbite, and straight facial profile. Analysis of the posttreatment and follow-up radiographs showed that the treatment outcomes remained stable seven years after active orthodontic treatment. Thus, although combined orthodontic and surgical treatment should be considered for patients with this skeletal malocclusion, this case report proves that well controlled orthodontic movement with the patient’s cooperation can be a valid alternative treatment, with good and stable outcomes for patients who refuse surgery.

## RESUMO

Paciente saudável, 15 anos de idade, com mordida aberta anterior, discrepância transversa e relação esquelética de Classe III, buscando tratamento ortodôntico não cirúrgico. O paciente foi tratado com extração de pré-molares associada a expansor Hyrax e mecânica de intrusão com elásticos verticais. Essa mecânica permitiu excelentes resultados faciais e oclusais. A oclusão final apresentou relação de molares e caninos em Classe I, sobremordida e sobressaliência ideais e perfil facial reto. A análise das radiografias pós-tratamento e o acompanhamento demonstraram que os resultados alcançados permaneceram estáveis sete anos após o tratamento ortodôntico ativo. Assim, embora o tratamento ortodôntico-cirúrgico combinado deva ser levado em consideração para pacientes com essa má oclusão esquelética, o presente relato de caso demonstrou que a movimentação ortodôntica bem controlada e com a colaboração do paciente se torna uma alternativa boa e estável para pacientes que recusam a cirurgia.

## INTRODUCTION

Anterior open bite is a complex and multifactorial vertical malocclusion, considered challenging to be treated due to its relapse rate.^1^ It can be caused by a combination of a wide range of factors, such as skeletal abnormalities, dentoalveolar abnormalities, respiratory and airway obstruction, neurological conditions and even by abnormal habits, like finger sucking, making its treatment more challenging.^2^ This malocclusion should be orthodontically treated before or during the peak of pubertal growth spurt.^3^ ^-^ ^6^ When treatment is delayed, the need for surgery increases and the treatment becomes more complex.^7-9^

The literature reports that the stability of open bite correction treated with the aid of a surgical procedure is approximately 75% to 85%^7^ ^,^ ^10^ ^-^ ^11^, but in orthodontically treated cases, the stability of the results is still questionable. The literature reports a relapse rate of molar intrusion from 22.88% to 30%.^12^ ^,^ ^13^ Beyond considerations of stability, the choice of treatment will depend on whether the deformity is skeletal, dental or both. 

The combination of an open bite, edge-to-edge transverse discrepancy and skeletal Class III, involving the three planes of space, in a patient after the peak of the growth spurt, is a challenging treatment and mostly involves orthognathic surgery or absolute anchorage, as miniplates or miniscrews.^2,3,14^ Most people, when having to choose between orthognathic surgery or a non-surgical camouflage treatment, prefer a nonsurgical treatment, because of the post-operative discomfort such as pain, edema and also the high costs of surgery. Nevertheless, camouflage should only be indicated as an option if the treatment outcomes will satisfy both the facial esthetics and the functional complaints of the patient.^15^ Facial esthetics is a critical issue that directly or indirectly affects self-esteem and social acceptance.^16^ ^,^ ^17^

Therefore, the aim of this case report was to describe a compensatory orthodontic approach of an anterior open bite, edge-to-edge transverse discrepancy and skeletal Class III in a patient in the final stage of the growth spurt, using premolars extraction, intrusion mechanics and maxillary expansion, and present its stability after 7 years.

## DIAGNOSIS AND ETIOLOGY

A healthy 15-year-old boy presented for orthodontic treatment with the chief complaint that his teeth were very protrusive. During the clinical examination, the patient showed mouth breathing, atypical swallowing and phonation (lingual interposition), and amelogenesis imperfecta on tooth #41. The patient had no previous medical or dental history.

**Figure 1:**
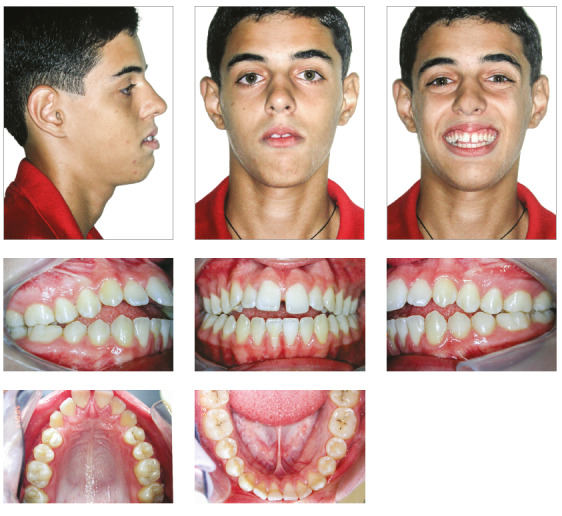
Pretreatment facial and intraoral photographs.

The patient presented a convex profile. His lips were incompetent at rest and his lower lip was hypertonic (Fig 1). The photographs indicated a facial asymmetry to the left and a gingival smile. There were no temporomandibular joint symptoms.

**Figure 2:**
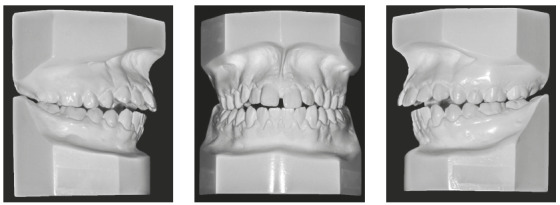
Pretreatment study casts.

Intraorally, he had a Class I molar relationship, an anterior open bite and edge-to-edge transverse discrepancy. The maxillary dental midline coincided with both the mandibular midline and the facial midline. The maxillary arch had an elliptical shape, while the mandibular arch was parabolic, with 0.5-mm crowding. His overjet and overbite were +6 mm and -4mm, respectively (Figs 1 and 2).

**Figure 3:**
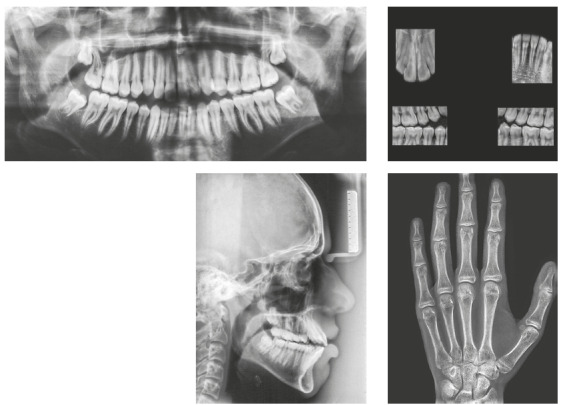
Pretreatment radiographs.

The panoramic radiograph showed the presence of all permanent teeth. The cephalometric analysis showed a Class III skeletal relationship (ANB = -1°, Wits = -2 mm), high mandibular plane angle (GoGn-SN = 38°) and increased lower facial height ratio (61%). The maxillary and mandibular incisors were protrusive (1.NA = 33°, 1-NA = 9 mm; 1.NB = 34°, 1-NB = 8 mm; IMPA = 98°) (Fig 3).

According to the maturation stage of cervical vertebrae on the lateral cephalometric radiograph and the hand and wrist radiograph^18^, the patient had passed his growth peak, but he still had a minimum period of residual growth (Fig 3).

## TREATMENT OBJECTIVES

The treatment objectives were to: (1) expand the maxilla to correct the transverse deficiency; (2) level and align the dental arches; (3) extract four premolars to retract the incisors; (4) obtain an ideal overbite and overjet, to establish correct anterior guidance; and (5) improve the facial profile.

## TREATMENT ALTERNATIVES

Orthodontic-surgical treatment could be considered the best treatment option for this patient, since skeletal discrepancy correction and establishment of an ideal occlusion and dental esthetics would be possible with this treatment. However, the patient and his parents refused orthognathic surgery, and the patient did not have any complaints regarding his face.

As an alternative to orthognathic surgery, a treatment with extraction of the first premolars combined with TADs to intrude the molars was considered, but even this minor surgical intervention was also refused.

The third option included a Hyrax expander, and extraction of the four first premolars combined with intrusion mechanics and vertical elastics. The patient was informed about the need for full cooperation and he agreed with this treatment plan.

## TREATMENT PROGRESS

**Figure 4:**
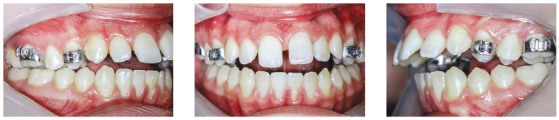
Intraoral photographs after maxillary expansion.

**Figure 5:**
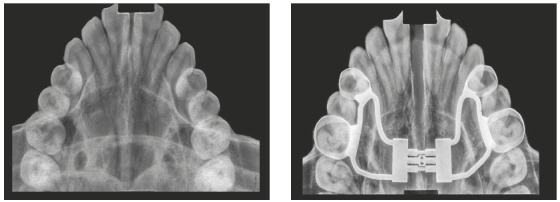
Occlusal radiographs before and after maxillary expansion.

Hyrax expander was placed at the initial part of treatment. The treatment protocol involved a ¼ turn twice a day for the first appliance (7-mm screw). Then, a new Hyrax appliance was installed (13-mm screw), and activated once a day. The expansion stopped when the edge-to-edge transverse discrepancy was overcorrected (Figs 4 and 5), and the expander was kept as a retainer for 6 months.

Then, fixed Edgewise appliances were bonded, to continue treatment. The four first premolars were extracted, and the arches were leveled and aligned with a series of stainless-steel round wires. After that, en-masse retraction was conducted with a 0.019 x 0.025-in stainless steel archwire. At this point, the patient also underwent extraction of the third molars. 

**Figure 6:**
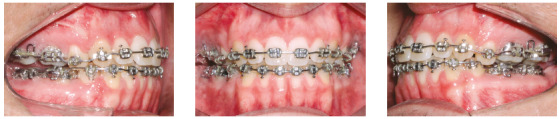
During orthodontic treatment with fixed appliances.

With all spaces closed, a 0.019 x 0.025-in stainless steel archwire with accentuated curve of Spee and tip-back bends was inserted in the maxillary arch, and a 0.019 x 0.025-in stainless steel archwire with reverse curve of Spee and tip-back bends was inserted in the mandibular arch (Fig 6). Vertical intermaxillary elastics (3/16-in) were used in the anterior region, with 150 g of force, allowing moderate mouth opening. The accentuated and the reverse curves of Spee, respectively on the maxillary and mandibular arches, along with anterior vertical elastics, had the function of promoting efficient vertical control, closing the anterior open bite and intruding the posterior teeth. The patient’s cooperation was excellent, facilitating the success of the mechanics employed. With the open bite correction, the archwires were segmented mesial to the molars, allowing for their extrusion.

After fixed appliance removal, the patient was instructed to wear an upper removable retainer with a tongue positioner and a lower bonded canine-to-canine retainer. The occlusal relationship was maintained after 7 years of retention.

## TREATMENT RESULTS

**Figure 7:**
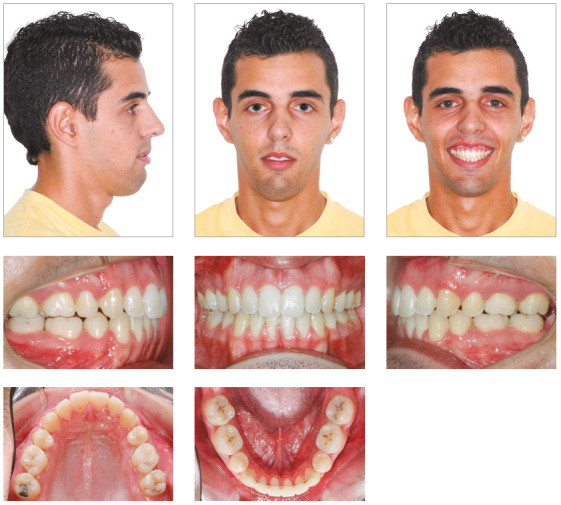
Posttreatment facial and intraoral photographs.

**Figure 8:**
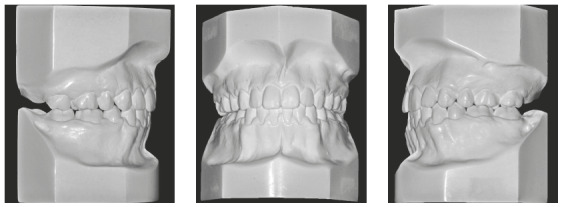
Posttreatment study casts.

Excellent facial and occlusal results were achieved with this non-surgical management, post-treatment photographs (Figs 7 and 8) show a Class I relationship and ideal overjet and overbite. The facial profile became straight, upper lip protrusion was improved and there was a slight increase of the lower facial height. Intraorally, the arch length and width deficiencies were eliminated, and a satisfactory tooth alignment was obtained. The patient’s compliance was very important for achieving these results.

**Figure 9:**
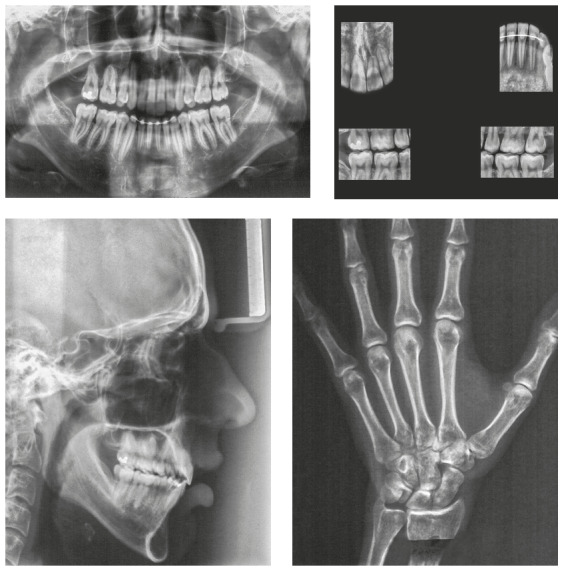
Posttreatment radiographs.

**Figure 10:**
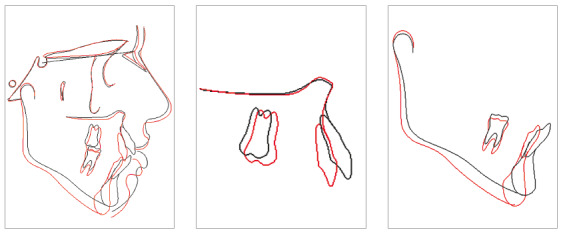
Superimposition of initial and final cephalometric tracings.

**Figure 11:**
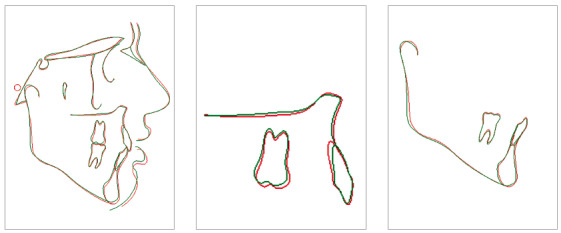
Superimposition of final and posttreatment cephalometric tracings.

In the panoramic radiograph, suitable root parallelism and moderate root resorption were noted (Fig 9). Cephalometric superimposition confirmed the mandible’s vertical growth pattern, and also presented a clockwise rotation of the palatal plane after treatment. Furthermore, the maxillary and mandibular incisors were retracted and extruded, while maxillary and mandibular molars moved mesially (Figs 10 and 11, and Table 1).

**Table 1:** Summary of Cephalometric Analysis

	Measurements	Normal	Pretreatment	Posttreatment	Follow-up
Skeletal pattern	SNA (degrees)	82	76	73	73
	SNB (degrees)	80	77	72.5	73
	ANB (degrees)	2	-1	0.5	0
	Wits (mm)	0 ± 2	-2	-0.5	-0.5
	SN-GoGn (degrees)	32	38	41	42
Dental pattern	1.NA (degrees)	22	33	24	23
	1-NA (mm)	4	9	7	7.5
	1.NB (degrees)	25	34	25	24
	1-NB (mm)	4	8	6	5.5
	1/1 interincisal angle (degrees)	130	112	129	130
	IMPA (degrees)	90	98	85	85

**Figure 12:**
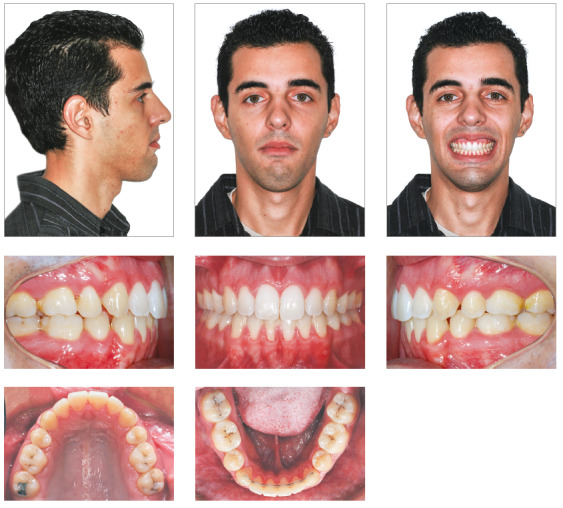
Posttreatment facial and intraoral photographs at 2 years follow-up.

**Figure 13:**
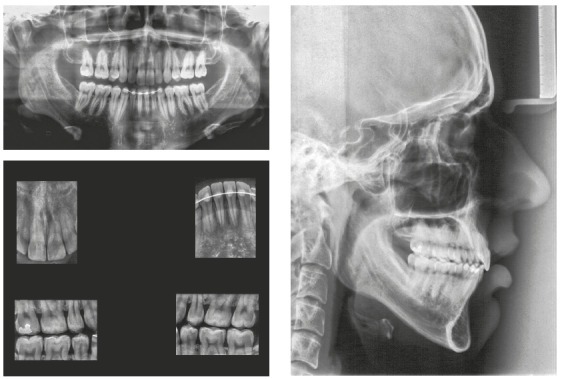
Posttreatment radiographs at 2 years follow-up.

At the follow-up appointments at 2 years and 7 years after treatment, stability of the dental and skeletal vertical dimension as well as the overjet and overbite was verified (Figs 11, 12 and 13). In addition, the root resorption remained stable. Despite the satisfaction of the patient after treatment, he still had vertical maxillary excess (gingival smile) and asymmetry to the left, issues that could only be corrected by orthognathic surgery.

**Figure 14:**
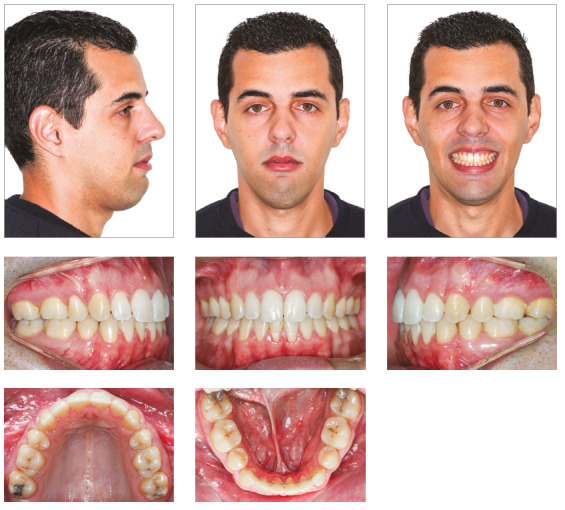
Seven-year posttreatment facial and intraoral photographs.

## DISCUSSION

Taking into account the limitations of orthodontic treatment, this patient would ideally be treated with an orthodontic-surgical management. This type of treatment has traditionally been the most effective way to solve a moderate to severe anterior open bite. However, surgery has complications and some patients may refuse it due to the invasive nature.^8^

A nonsurgical plan usually requires a longer treatment time, increases the difficulty of treatment and may compromise the long term stability. The skeletal discrepancies are camouflaged as much as possible to try to satisfy the patient’s esthetic and functional complaints.^19^ The chief complaint of this patient was that his teeth were protrusive. This 15-year-old boy presented with edge-to-edge transverse discrepancy, anterior open bite and Class III skeletal relationship. It was a great challenge to plan his treatment in all three dimensions.

Correcting his transverse discrepancy after his pubertal growth peak was the first challenge, since the parents refused any invasive treatment. Many authors would not suggest a Hyrax expansion^5^ at this age or would consider the use of MARPE to obtain expansion.^12^ However, the patient’s occlusal radiographs (Fig 5) showed a gap in the median palatal suture, confirming the success of the skeletal expansion. 

Considering that the maxilla is frequently displaced downward and forward during maxillary expansion, a slight mandible clockwise rotation is expected.^20^ This change was observed in this patient, where the ANB angle changed from -1^o^ to 0.5^o^, improving the Class III skeletal relationship but also increasing the anterior facial height.

For the control of anterior facial height, the most frequent approach is the intrusion of the posterior teeth, that can be accomplished with the aid of a skeletal anchorage such as dental implants, miniscrews and miniplates, but they require surgery.^21^ ^-^ ^23^ In this case report, to correct the anterior open bite, the bicuspid extraction was indicated to allow for precise and accurate intrusion mechanics of the posterior teeth. 

Both the accentuated upper curve of Spee and the reverse lower curve of Spee with tip back bends were inspired by the multiloop Edgewise archwire therapy described by Kim et al.,^24^ but we chose not to insert the multiloops due to the impaired aesthetics and discomfort to the patient. Regardless, these mechanics still require excellent compliance for treatment success and the force used in the elastics mechanics must be higher than the intrusion force used in the archwires, otherwise the mechanics will worsen the open bite. 

The incisors retraction and almost no posterior teeth extrusion allowed for improvement of the facial profile and the achievement of a good occlusion. If the treatment option had been the use of Class III elastics, the posterior teeth extrusion would be inevitable, worsening the vertical problem and probably compromising the final results.

Although the stability of open bite correction is questionable, after 7-year follow-up in this case, the occlusion remained stable. This long-term stability is probably due to: the success of the skeletal expansion of the maxillary arch; the control of the mechanics, not allowing the posterior teeth to extrude; the improvement of the patient’s tongue posture after he was referred to a speech therapist; and the careful attention during the retention phase with the use of a tongue positioner along with very good compliance regarding its use by the patient.

## CONCLUSION

The combination of premolars extraction, Hyrax expander and intrusion mechanics with vertical elastics was demonstrated to be an effective treatment for this patient. The patient’s follow-up records show high stability of this treatment.
